# Clinicopathological Profile of Appendicular Disease in Children: A Tertiary Health Care Center Study

**DOI:** 10.7759/cureus.44697

**Published:** 2023-09-05

**Authors:** Saugata Ray, Umesh K Gupta, Gautam Prakash, Shesh Kumar

**Affiliations:** 1 Department of Surgery, Midnapore Medical College and Hospital, Midnapore, IND; 2 Department of Pediatric Surgery, Uttar Pradesh University of Medical Sciences, Etawah, IND; 3 Department of Pediatric Surgery, Indira Gandhi Institute of Medical Sciences, Patna, IND; 4 Department of General Surgery, Uttar Pradesh University of Medical Sciences, Etawah, IND

**Keywords:** perforated appendicitis, gangrenous appendicitis, appendicitis, appendectomy, pediatric appendicitis

## Abstract

Background

Acute appendicitis (AA) is the most common surgical emergency worldwide. Delay in diagnosis of disease often leads to serious complications such as perforation appendicitis (PA) and gangrenous appendicitis (GA).

Aims and objectives

The purpose of the study is to document clinicopathological outcomes in pediatric age group patients in a tertiary health care center.

Material and method

This study was a prospective observation study of 50 patients with pediatric appendicitis who had undergone emergency appendectomy from January 2022 to December 2022. All pediatric patients below 15 years of age with a diagnosis of AA were included. Institute ethical permission was granted before the study, and parent consent was taken for the surgery and also for inclusion in the study. After proper resuscitation, all patients underwent appendectomy, and necessary specimens were sent for histological examination. Based on histopathology reports, all patients were classified into four groups: AA, PA, GA, and normal appendix (NA).

Results

Out of 50 patients, 33 (66%) patients were males and 17 (34%) patients were females. The mean age of the patients was 10.22 ± 2.73 years. The mean age of AA, PA, GA, and NA patients were 10.25 ± 2.6 years, 9.78 ± 2.99 years, 10.00 ± 4.6 years, and 12.00 ± 2.8 years, respectively. The mean duration of symptoms at the time of hospital admission was 2.42 ± 0.97 days for histopathologically proven AA patients, 4.67 ± 2.1 days for GA patients, 2.8 ± 0.83 for PA patients, and one day for NA patients. Overall clinical presentation was right iliac fossa (RIF) pain in 36 (72%) patients, migration of pain in 31 (62%) patients, anorexia in 37 (74%) patients, nausea and vomiting in 43 (86%) patients fever in 26 (52%) patients, RIF tenderness in 50 (100%) patients, rebound tenderness in 39 (78%) patients, guarding in 19 (38%) patients, Psoas's sign in nine (18% patients), and Rovsing's sign in 19 (38%) patients. On histopathological examination of the sent specimen, AA was found in 36 (72%) patients, PA was found in nine (18%) patients, GA was found in three (6%) patients, and NA was found in two (4%) patients. Wound infection was the most common complication and was found in five (10%) patients. The average duration of hospital stay for AA, PA, GA, and NA was 4.33 ± 1.04 days, 9.56 ± 4.2 days, 12.33 ± 8.5 days, and 3.50 ± 0.71 days, respectively.

Conclusion

The appendicular disease is common in teenage male children. Fever, dehydration, and rebound tenderness at the RIF are clinically significant findings. Duration of symptoms at the time of diagnosis, post-appendectomy complication, and duration of hospital stay significantly correlated with histopathological findings.

## Introduction

Acute appendicitis (AA) is one of the most common causes of abdominal pain in childhood; around 20% to 30% of children have abdominal pain. Approximately 50% of children have benign, self-limiting, non-speciﬁc abdominal pain, as well as allergy, infection, mesenteric adenitis, transient intussusception, and so on [1]. The lifetime risk of AA in Western countries is approximately 7%, which is signiﬁcantly higher than that in low socioeconomic countries [1]. AA can occur at any age but is most commonly presented around 10-18 years of age [1]. Appendicitis is uncommon in very young children. In earlier childhood, atypical presentation of appendicular disease causes diagnostic challenges. Anatomical variation in the location of the tip of the appendix and pathophysiological differences may cause a variety of clinical presentations such as appendicular perforation, leading to localized abscess formation, generalized peritonitis, and sepsis [2]. Misdiagnosed case of appendicitis ranges from approximately 70% to 100% in children below two years of age to 28% to 57% in 2- to 12-year-old children, while reaching less than 15% in adolescents [2,3]. Up to 15% patients with appendicular disease were seen two to three times by emergency department clinicians before appendicitis diagnosis [3].

## Materials and methods

**Figure 1:**
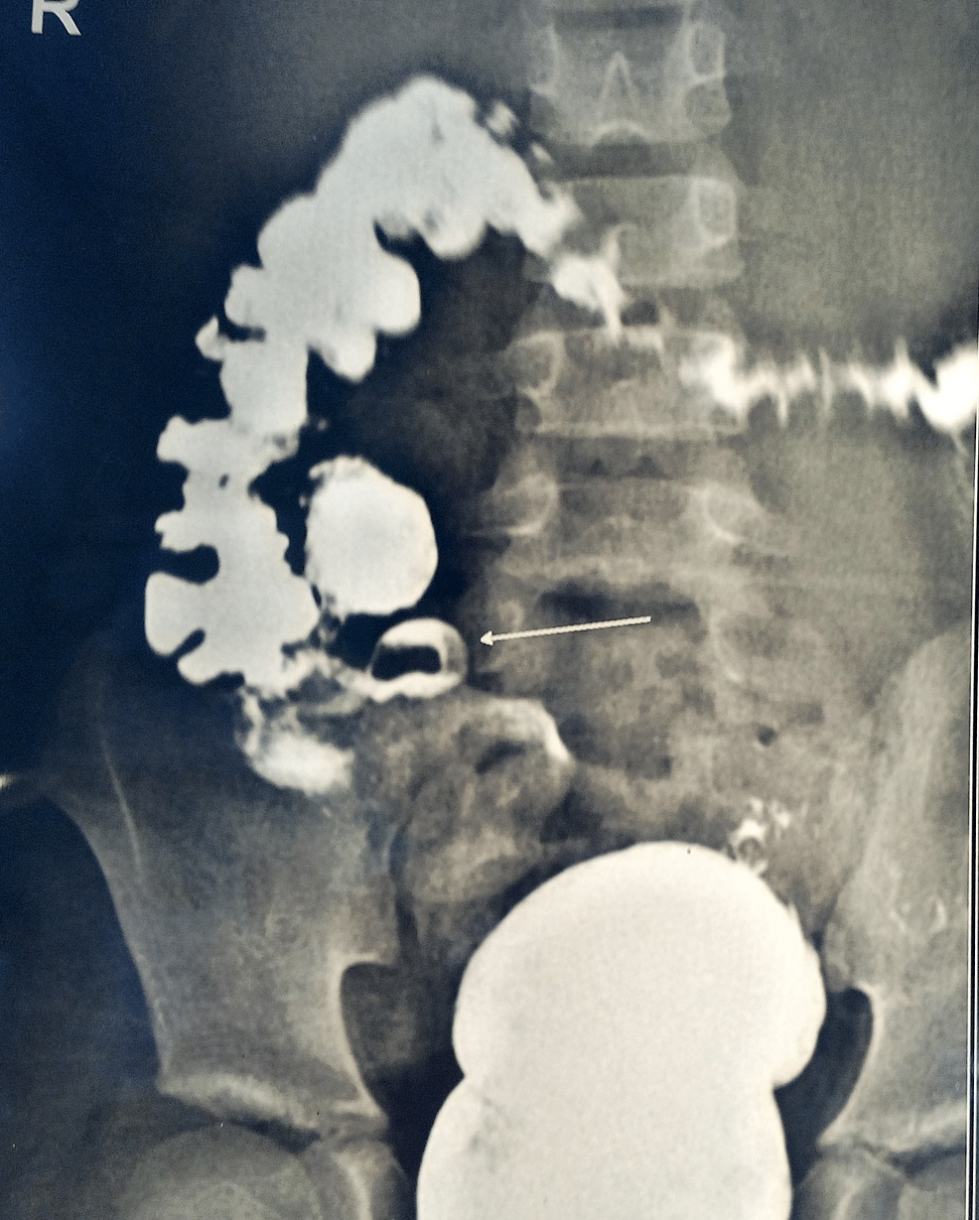
Barium meal follow-through showing radiolucent fecaliths inside the appendix (white arrow).

**Figure 2:**
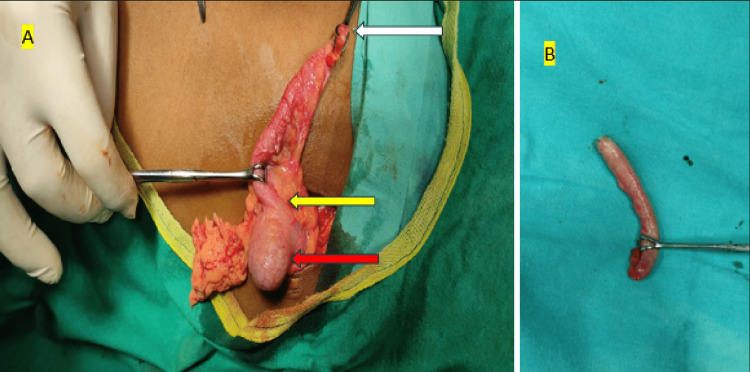
(A) Intraoperative image of appendectomy. (B) Post-appendectomy specimen of the appendix. (A) White arrow shows the tip of the appendix, yellow arrow shows the base of the appendix, and red arrow shows caecum at the level of the ileocecal junction.

This is a prospective observational study of 50 children who presented to the tertiary health care center of the Pediatric Surgery, a unit of the Department of General Surgery, Midnapore, West Bengal, India, from January 2022 to December 2022. During one year of the study period, patients up to 14 years of age whose parents gave necessary consent for participation in the study and surgery were included in the study. Patients older than 14 years and patients having incomplete data were excluded. Ethical clearance was obtained from the ethical committee of the institute before conducting the study. Patients were evaluated for the clinical signs and symptoms of appendicular disease. Necessary investigations such as complete blood count, liver function test, renal function test, ultrasonography, and others, if needed (Figure 1), were conducted. After proper resuscitation and injectable broad-spectrum antibiotic, all patients underwent appendectomy (Figure 2), and necessary specimens were sent for histopathological examination. Based on histopathology reports, all patients were classified into four groups: AA, perforated appendicitis (PA), gangrenous appendicitis (GA), and normal appendix (NA). Demographic data, clinical symptoms and signs of illness, and biochemical, radiological, intraoperative, and histological findings were noted. Data were compiled on an Excel spreadsheet and evaluated using suitable software for significant findings. The chi-square test and non-parametric test were applied for categorical data. Differences were considered significant when the p-value was <0.05.

## Results

In our study of 50 patients, 33 (66%) were males and 17 (34%) were females. The youngest patient was four years old, and the oldest patient was 14 years old. The mean age of the study patients was 10.22 ± 2.73 years. The mean age of the male and female patients was 10.42 ± 2.67 years and 9.82 ± 2.88 years, respectively. On histopathological examination of the specimen sent post-appendectomy, AA was found in 36 (72%) patients, of which 24 (66.7%) were males and 12 (33.3%) were females, PA was found in nine (18%) patients, of which six (66.7%) were males and three (33.3%) were females, GA was found in three (6%) patients, of which all were males, and NA was found in two (4%) patients, of which all were females (Table 1). The mean age of AA, PA, GA, and NA patients was 10.25 ± 2.6 years, 9.78 ± 2.99 years, 10.00 ± 4.6 years, and 12.00 ± 2.8 years, respectively.

**Table 1:** Distribution of patients according to age group, sex, and histopathological finding in appendicular disease.

Age group	Sex of patients		Histopathological finding			
	Male	Female	Acute appendicitis	Perforated appendicitis	Gangrenous appendicitis	Normal appendix
<5 years	1	1	1	1	0	0
5-10 years	14	9	17	4	1	1
11-14 years	18	7	18	4	2	1
Total	33	17	36	9	3	2

Four (11.1%) patients of AA were admitted within 24 hours, 19 (52.8%) patients were admitted between 24 and 48 hours, nine (25%) patients were admitted between 48 and 72 hours, and four (11.1%) patients were admitted after 72 hours from onset of symptoms. In the case of PA, four (44.4%) patients were admitted between 24 and 48 hours, three (33.3%) patients were admitted between 48 and 72 hours, and two (22.2%) patients were admitted after 72 hours from onset of symptoms. In the case of GA, one (33.3%) patient was admitted between 48 and 72 hours, two (66.7%) patients were admitted after 72 hours from the onset of pain, and none of the patients was admitted before 48 hours of symptoms. In the case of histopathologically proven NA, all patients were admitted within the first 24 hours (Table 2). The mean duration of symptoms at the time of hospital admission was 2.42 ± 0.97 days for histopathologically proven AA, 4.67 ± 2.1 days for GA, 2.8 ± 0.83 for PA, and one day for NA. The duration of symptoms at the time of hospital admission and histopathological findings were statistically significant (Pearson’s chi-square test=38.15, degree of freedom=15, p<0.001) (Table 2).

**Table 2:** Distribution of patients according to the duration of symptoms at the time of admission with histopathological findings in appendicular disease.

Duration of symptoms at the time of admission (hours)	Acute appendicitis (n=36)	Perforated appendicitis (n=9)	Gangrenous appendicitis (n=3)	Normal appendix (n=2)	P-value
< 24 hours	4	0	0	2	<0.001
>24-48 hours	19	4	0	0	
>48-72 hours	9	3	1	0	
>72 hours	4	2	2	0	

Symptoms in the histopathologically proven AA cases were pain in the right iliac fossa (RIF) in 24 (66.7%) patients, migration of pain in 21 (58.3%), anorexia in 23 (63.9%) patients, nausea and vomiting in 31 (86%) patients, and fever in 14 (38.9%) patients. Symptomatology in cases of PA was RIF pain and migration of pain in seven (77.8%) patients, anorexia and fever in nine (100%) patients, and nausea and vomiting in eight (88.9%) patients. Symptomatology in cases of GA was RIF pain, anorexia, nausea and vomiting, and fever in three (100%) patients, while two (66.7%) patients presented with migration of pain. Symptomatology in histologically proven NA cases was RIF pain and anorexia in two (100%) patients, and migration of pain and nausea, and vomiting were present in one (50%) patient (Table 3). In our study, symptomatology was correlated to histological findings, and data showed significance for fever (Pearson’s chi-square test=15.72, degree of freedom=3, p<0.001), while RIF pain (p=0.46), migration of pain (p=0.73), anorexia (p=0.08, and nausea and vomiting (p=0.44) were not significant (Table 3).

**Table 3:** Distribution of clinical symptoms and histopathological findings in appendicular disease.

	Histopathological finding				P-value
Symptomatology	Acute appendicitis (n=36)	Perforated appendicitis (n=9)	Gangrenous appendicitis (n=3)	Normal appendix (n=2)	
Right iliac fossa pain	24	7	3	2	0.46
Migration of pain	21	7	2	1	0.73
Anorexia	23	9	3	2	0.08
Nausea and vomiting	31	8	3	1	0.44
Fever	14	9	3	0	0.001

Clinical signs in the histopathological finding of AA cases were elevated temperature in seven (19.4%) patients, dehydration in 11 (30.6%) patients, RIF tenderness in all (n=36) patients, rebound tenderness in 27 (75%) patients, guarding in eight (22.2%) patients, Psoas's sign in three (8.3%) patients, and Rovsing's sign in seven (19.4%) patients. Clinical signs in PA cases were elevated temperature, dehydration, and guarding in eight (88.9%) patients; RIF tenderness, rebound tenderness, and Rovsing's sign were presented in all (n=9) patients, while Psoas's signs were presented in three (33.3%) patients. Clinical signs in GA cases were elevated temperature, dehydration, RIF tenderness, rebound tenderness, guarding, positive Rovsing's sign, and positive Psoas's sign, which were presented in all (n=3) patients. Clinical signs in histopathologically proven NA cases were RIF tenderness in all (n=2) patients, while none of the cases was with elevated temperature, dehydration, rebound tenderness, guarding, Psoas's sign, and Rovsing's sign (Table 4). In our study, clinical signs were correlated to histological findings, with data showing significance for dehydration (Pearson’s chi-square test=15.4, degree of freedom=3, p<0.002) and rebound tenderness (Pearson’s chi-square test=107, degree of freedom=3, p<0.014), while temperature (p=0.000), RIF tenderness (non-statistical), guarding (p=0.000), Psoas’ sign (p=0.000), and Rovsing’s sign (p=0.000) were not significant (Table 4).

**Table 4:** Distribution of clinical signs and histopathological findings in appendicular disease. NS, non-statistical

	Histopathological finding				P-value
Clinical signs	Acute appendicitis (n=36)	Perforated appendicitis (n=9)	Gangrenous appendicitis (n=3)	Normal appendix (n=2)	
Temperature	7	8	3	0	0.000
Dehydration	11	8	3	0	0.002
Right iliac fossa tenderness	36	9	3	2	NS
Rebound tenderness	27	9	3	0	0.014
Guarding	8	8	3	0	0.000
Psoas’s sign	3	3	3	0	0.000
Rovsing’s sign	7.	9	3	0	0.000

Total leukocyte count (TLC) was >10,000 cells/mm^3^ in 33 (91.7%) AA patients, nine (100%) PA patients, and three (100%) GA patients, while none of the NA patients had TLC>10,000 cells/mm^3^. The mean TLC in AA, PA, GA, and NA was 12,420 ± 2,377 cells/mm^3^, 22,922 ± 8,757 cells/mm^3^, 31,733 ± 9,940 cells/mm^3^, and NA 9300 ± 707 cells/mm^3^, respectively. Absolute neutrophil count (ANC) was >5,000 cells/mm^3^ in 34 (94.4%) AA patients, nine (100%) PA patients, and three (100%) GA patients, while none of the NA patients had ANC>5,000. The mean ANC in AA, PA, GA, and NA was 9,935 ± 2,296 cells/mm^3^, 19,152 ± 7,231 cells/mm^3^, 233 ± 8,794 cells/mm^3^, and 5,700 ± 1,697 cells/mm^3^, respectively. The left shift of neutrophil >75% were found in 28 (77.8%) AA patients, nine (100%) PA patients, and three (100%) GA patients, while none of the NA patients had left shift of neutrophil>75%. The mean left shift of neutrophils>75% was 79.2% ± 6.2% in AA patients, 83.8% ± 2.8% in PA patients, 82.9% ± 5.4% in GA patients, and 60.7% ± 13.7% in NA patients. Increased C-reactive protein (CRP) (>3 mg/dL) was found in all cases of AA, PA, and GA, while none of the NA patients had increased CRP (>3 mg/dL). The mean CRP in AA, PA, GA, and NA was 12.5 ± 7.1 mg/dL, 58.3 ± 20.9 mg/dL, 69.7 ± 26.5 mg/dL, and 7.0 ± 1.4 mg/dL, respectively. In our study, elevated erythrocyte sedimentation rate (ESR) (>20 mm/hr) was found in 26 (72.2%) AA patients, eight (88.9%) PA patients, and three (100%) GA patients, while none of the NA patients had elevated ESR (>20 mm/hr) (Table 5).

**Table 5:** Distribution of laboratory markers and histopathological findings in appendicular disease. TLC, total leukocyte count; ANC, absolute neutrophil count; CRP, C- reactive protein; ESR, erythrocyte sedimentation rate

Laboratory investigation	Acute appendicitis (n=36)	Perforated appendicitis (n=9)	Gangrenous appendicitis (n=3)	Normal appendix (n=2)
TLC>10,000 cells/mm^3^	33	9	3	0
ANC>5,000 cells/mm^3^	34	9	3	0
Neutrophil>75%	28	9	3	0
CRP>3 mg/dL	36	9	3	2
ESR>20 mm/hr	26	8	3	0

Postoperatively, wound infection was found in three (8.3%) AA patients, one (11.1%) PA patients, and one (33.3%) GA patients. Pelvic abscess was seen in three (33.3%) PA patients, while none of the patients with PA, GA, and NA presented with pelvic abscess. Postoperative intestinal obstruction was found in one patient each of PA and GA (Table 6). No mortality was recorded during the postoperative period. In our study, postoperative complications correlated with histological findings, and data showed significance (Pearson’s chi-square test=27.7, degree of freedom=9, p<0.001, with a likelihood ratio of 21.5) (Table 6).

**Table 6:** Distribution of postoperative complications with histopathological findings in appendicular disease.

Postoperative complications	Histopathological finding				P-value
	Acute appendicitis (n=36)	Perforated appendicitis (n=9)	Gangrenous appendicitis (n=3)	Normal appendix (n=2)	
Wound infections	3	1	1	0	<0.001
Pelvic abscess	0	3	0	0	
Intestinal obstruction	0	1	1	0	
Absent	33	4	1	2	

The duration of hospital stay (DOHS) was less than five days in 28 (77.8%) patients with AA and all (n=2) cases of histopathologically proven NA. In our study, overall, 30 (60%) patients were admitted for less than five days, 16 (32%) patients were admitted for 5-10 days, and four (8%) patients were admitted for more than 10 days (Table 7). The average DOHS for AA, PA, GA, and NA was 4.33 ± 1.04 days, 9.56 ± 4.2 days, 12.33 ± 8.5 days, and 3.50 ± 0.71 days, respectively. In our study, the DOHS correlated to histological findings, and data were significant (Pearson’s chi-square test=71.07, degree of freedom=33, p<0.001, with a likelihood ratio of 51.24) (Table 7).

**Table 7:** Distribution of patients according to the duration of hospital stay and histopathological findings in appendicular disease.

Duration of hospital stay	Histopathological finding				P-value
	Acute appendicitis (n=36)	Perforated appendicitis (n=9)	Gangrenous appendicitis (n=3)	Normal appendix (n=2)	
<5 days	28	0	0	2	0.0001
5 days to 10 days	8	6	2	0	
>10 days	0	3	1	0	

## Discussion

In this study, the incidence of AA, PA, and GA on histopathological examination post-appendectomy was 72%, 18%, and 6%, respectively, which was similar to the findings by Salo et al. [4], Hernandez et al. [5] and Singh et al. [6]. In our study, the rate of a negative appendectomy was 4%, similar to the findings by Benito et al. [7] and Kosloske et al. [8]. The rate of AA and PA was three times more common in males than females in our study, which was similar to the findings by Salo et al. [4], Hernandez et al. [5], and Singh et al. [6]. In our study, PA in those below five years of age was found in 11.1%, which is contrary to that reported by Singh et al. [6]. Singh et al. found 100% of appendicular perforation in those below five years of age [6]. In our study, the mean age of PA (9.78 ± 2.99 years) and GA (10.00 ± 4.6 years) patients were lower than AA (10.25 ± 2.6 years) patients, which was similar to the findings by Hernandez et al. [5] and Cayrol et al. [9].

In our study, 58% of patients with appendicular disease were admitted to the hospital within the first 48 hours and 42% of patients were admitted after 48 hours of illness, comparable with the study by Murthy and Panda [10]. Murthy and Panda found that 40% of patients were admitted to the hospital within 48 hours of illness and 60% were admitted after 48 hours of illness [10]. The late presentation of symptoms (>24 hours) at the time of admission in our study was in 92% of patients, which was contrary to the study by Singh et al. [6] and Bachur et al. [11]. Singh et al. [6] and Bachur et al. [11] found the late presentation of symptoms at the time of admission in 36% to 46%, while early presentation was found in 54% to 64%. A higher rate of misdiagnosis of AA and delayed referral to higher health care centers may be the causes of late presentation of symptoms.

RIF pain and RIF tenderness were the most common clinical features in our study and were found in all patients, similar to the findings by Cayrol et al. [9], Colvin et al. [12], Santillanes et al. [13], and Bachur et al. [11]. In our study, clinical symptoms in histopathologically proven AA patients were RIF pain (66% of patients), migration of pain (58% of patients), anorexia (64% of patients), vomiting (86% of patients), and fever (38% of patients), which is comparable to Colvin et al.’s study [12]. The study by Colvin et al. found RIF pain in 74% of patients, migration of pain in 39% of patients, anorexia in 75% of patients, vomiting in 66%% of patients, and fever in 47% of AA patients [12]. In our study, clinical signs in AA were RIF tenderness (96% of patients), rebound tenderness (78% of patients), guarding (38% of patients), psoas sign (18% of patients), and Rovsing’s sign (38% of patients), which was comparable to Colvin et al.’s study [12]. In the study by Colvin et al., RIF tenderness was found in 47% of AA patients, rebound tenderness in 65% of AA patients, guarding in 65% of AA patients, psoas sign in 30% of AA patients, and Rovsing’s sign in 26% of AA patients [12]. In our study, clinical symptoms in histopathologically proven PA were RIF pain (78% of patients), migration of pain (78% of patients), anorexia (100% of patients), nausea and vomiting (88% of patients), and fever (100% of patients), which was comparable to the findings by Nelson et al. [14]. In the study by Nelson et al., anorexia, vomiting, and fever were reported in 91%, 86%, and 83% of cases, respectively [14]. Clinical signs in histopathologically proven PA were RIF tenderness (100% of patients), rebound tenderness (100% of patients), guarding (88% of patients), psoas signs (33% of patients), and Rovsing’s sign (100% of patients), which was comparable to the findings by Nelson et Al. [14]. In the study by Nelson et al., rebound tenderness was found in 72% of patients and guarding was found in 87% of patients. In our study, clinical symptoms in histologically proven GA were RIF pain (100% of patients), migration of pain (66.7% of patients), nausea and vomiting (100% of patients), and fever (100% of patients). This was comparable with the findings by Cayrol et al. [9], who found RIF pain in 34.5% of patients, migration of pain in 32.1%, vomiting in 75.8%, and fever in 51.7% of patients. Clinical signs in our study were abdominal tenderness and rebound tenderness, which were present in 100% of patients, while in the study by Cayrol et al. [9], abdominal tenderness and rebound tenderness were present in 79.3% and 58.6% of patients, respectively.

In our study, the TLC count, ANC count, CRP, and mean percentage of the neutrophil count were elevated in PA and GA than in AA, which is similar to the findings by Hernandez et al. [5] and Cayrol et al. [9] study. In our study, the mean values of TLC count, ANC count, CRP, and mean percentage of the neutrophil count were observed much higher in PA than in AA, which was similar to the findings by Hernandez et al. [5], Cayrol et al. [9], Sack et al. [15], and Siddique et al. [16].

In our study, wound infection was the most common complication, followed by pelvic abscess, and the complication rate was much higher in PA and GA than in AA. No mortality was recorded during the postoperative period. The complication rate in our study was similar to others [5,8,16]. The average DOHS was found higher in AP than in AA, which was similar to other studies [5,13,16].

## Conclusions

Appendicular disease is common in teenage children, and the male sex is commonly affected. Very young children are often misdiagnosed and present with serious complications and poor outcomes. There was no mortality in this study. Fever, dehydration, and rebound tenderness at the RIF were significant findings. Duration of symptoms at the time of diagnosis, post-appendectomy complication, and DOHS significantly correlated with histopathological findings.
